# Effects of Proton Pump Inhibitors on Cardiovascular Events and Inflammatory Factors in Patients With Upper Gastrointestinal Bleeding Undergoing Dual Antiplatelet Therapy

**DOI:** 10.7759/cureus.59925

**Published:** 2024-05-08

**Authors:** Farhan Aslam, Afaq Naeem, Emad Munir, Hamna Jabeen Ashraf, Bilawal Ali, Bilal Qammar, Maham Farooq, Sami Ullah, Sumbal Jawad

**Affiliations:** 1 Orthopedics, Sir Ganga Ram Hospital, Lahore, PAK; 2 Internal Medicine, Shalamar Hospital, Lahore, PAK; 3 Cardiology, Shalamar Hospital, Lahore, PAK; 4 Internal Medicine, DHQ Teaching Hospital, Dera Ghazi Khan, PAK; 5 Public Health Practices and Administration, Institute of Public Health, Lahore, PAK; 6 Medicine, DHQ Teaching Hospital, Gujranwala, PAK

**Keywords:** inflammatory factors, cardiovascular events, upper gastrointestinal bleeding, dual antiplatelet therapy, proton pump inhibitors

## Abstract

Introduction: Dual antiplatelet therapy (DAPT), vital post-percutaneous coronary intervention (PCI) to prevent cardiovascular events (CVEs) via aspirin and P2Y12 receptor antagonists, faces controversy when combined with proton pump inhibitors (PPIs) due to potential impacts on bleeding risk and antiplatelet efficacy, prompting the need for further research to determine optimal co-administration practices. This work evaluated the effects of PPIs on CVEs and inflammatory factors in patients with upper gastrointestinal bleeding (UGIB) undergoing DAPT after PCI.

Materials and methods: The data of 166 patients who underwent PCI and developed UGIB while on DAPT from April 2021 to April 2023 were retrospectively analyzed. The patients were rolled into two groups: those who received PPI treatment and those who did not, namely, the PPI and non-PPI group, respectively. Clinical data from these patients was analyzed, intending to provide relevant theoretical evidence for clinical practice. Furthermore, the occurrence of CVEs and the levels of inflammatory factors of patients in all groups were statistically analyzed.

Results: Melena was the most common clinical symptom observed in all UGIB patients. The incidence of CVEs in the PPI group was not greatly different from that in the non-PPI group (*P*>0.05). The distribution of CVEs occurrence among different PPI drugs also exhibited no obvious difference (*P*>0.05). The PPI group exhibited greatly lower C-reactive protein (CRP) and tumor necrosis factor α (TNF-α) based on the non-PPI group (*P*<0.05).

Conclusion: Melena was the most frequent clinical manifestation in UGIB patients. The use of PPIs did not increase the risk of CVEs, and different PPI drugs did not affect the occurrence of CVEs. Furthermore, PPIs lowered CRP and TNF-α levels in serum of these patients.

## Introduction

Dual antiplatelet therapy (DAPT) is commonly applied after percutaneous coronary intervention (PCI) to prevent cardiovascular events (CVEs). It involves the simultaneous use of two antiplatelet drugs to reduce platelet aggregation and prevent thrombus formation [1]. Clinically, aspirin and P2Y12 receptor antagonists such as clopidogrel, prasugrel, or ticagrelor are commonly used [2]. The principle of DAPT is based on the crucial role of platelets in thrombus formation. Platelets are cell fragments in the blood that rapidly aggregate at the site of a blood vessel injury, forming platelet aggregates (thrombi) to minimize bleeding [3]. However, in certain disease states like coronary artery disease, excessive platelet aggregation can lead to the formation of intravascular thrombi, which may trigger CVEs such as myocardial infarction (MI) or stroke [4]. By using both aspirin and P2Y12 receptor antagonists, platelet inhibition is achieved. Aspirin inhibits the synthesis of thromboxane A2, a platelet-aggregating substance, thus blocking platelet activation and aggregation [5]. P2Y12 receptor antagonists, on the other hand, block the binding of adenosine diphosphate (ADP) (a signaling molecule that promotes platelet aggregation) to the P2Y12 receptors on the platelet surface, reducing platelet activation and aggregation. DAPT can also cause some side effects, with the most common being an increased risk of bleeding, including gastrointestinal bleeding and other bleeding events [6].

Proton pump inhibitors (PPIs), commonly used to treat gastric acid-related disorders including gastric ulcers, gastroesophageal reflux disease (GERD), and Barrett’s esophagus, not only reduce stomach acid production [7] but also modulate inflammatory factors within the gastric mucosa, potentially enhancing their therapeutic effects. PPIs work by inhibiting the activity of the proton pump (also known as the proton/acid-secreting ATPase) on the gastric mucosa, thereby reducing the production and secretion of stomach acid. The proton pump is an intracellular organelle responsible for secreting stomach acid from the gastric lumen into the gastric wall. It promotes the production of stomach acid by linking intracellular hydrogen ions (protons) with the pathway of acid secretion. The mechanism of action of PPIs involves two key steps. First, these drugs enter the stomach and are activated by gastric acid [8]. Then, they enter the cells of the gastric wall and bind to the proton pump enzyme, blocking its association with protons, thus inhibiting the secretion of stomach acid. This blocking action is reversible; the binding of the drug with the proton pump enzyme is reversible, allowing the drug to gradually be released from the cells after a period of time [9].

However, there is still controversy regarding the real clinical impact of PPIs in patients undergoing DAPT with PCI. Some studies have suggested that the use of PPIs is associated with a higher incidence of CVEs [10]. Furthermore, PPIs may alter the gastric acid environment, affecting the absorption and efficacy of P2Y12 receptor antagonists. Some studies suggest that the benefits of PPIs in reducing the risk of upper gastrointestinal bleeding (UGIB) may outweigh their impact on antiplatelet effects, and therefore, it is still recommended to use them in combination in patients at high risk of bleeding [11,12]. On the other hand, the use of PPIs in low-risk bleeding patients may have a negative impact on the clinical efficacy of DAPT. As a result, further research and clinical trials are needed to clarify whether patients should be co-administered with PPIs or not.

Objective

The objective of this work is to assess the impact of PPIs on CVEs and inflammatory factors in patients with UGIB undergoing DAPT following PCI, aiming to offer theoretical evidence for clinical practice.

## Materials and methods

Study design

From April 2021 to April 2023, a retrospective study was conducted, involving 166 patients who underwent PCI while on DAPT, resulting in UGIB.

Study subjects

Diagnostic criteria for UGIB were elucidated as follows: mild bleeding was characterized by a positive fecal occult blood test (FOBT) and a mild decrease in hemoglobin levels, while severe bleeding was defined by a hemoglobin drop of ≥ 50 g/L, severe hypotension necessitating transfusion of ≥ 200 ml blood, or the utilization of intravenous vasopressors or surgical intervention.

Patients included in the study had to meet specific criteria: Firstly, all enrolled individuals were diagnosed in accordance with the diagnostic criteria outlined by the European Society of Cardiology (ESC)/American College of Cardiology (ACC) in 2018, relying on clinical examination, electrocardiography, cardiac enzyme analysis, and coronary angiography. Secondly, patients underwent PCI for the first time and were capable of adhering to the prescribed regular DAPT regimen, consisting of aspirin 100 mg/d and clopidogrel 75 mg/d, as advised by medical professionals. Additionally, patients had to be at least 18 years old, present stable vital signs, maintain alert consciousness, and meet the diagnostic and grading criteria for UGIB.

Exclusion criteria encompassed severe liver or kidney dysfunction, immunological diseases, long-term use of oral anticoagulant drugs for conditions such as atrial fibrillation or pulmonary embolism, multi-organ diseases with an anticipated life expectancy of less than one year, prolonged intake of non-steroidal anti-inflammatory drugs (NSAIDs) for conditions like rheumatoid arthritis, pregnancy or breastfeeding, age below 18 years, and individuals lost to follow-up or lacking significant clinical data.

Methods for surgery and detection

**Figure 1:**
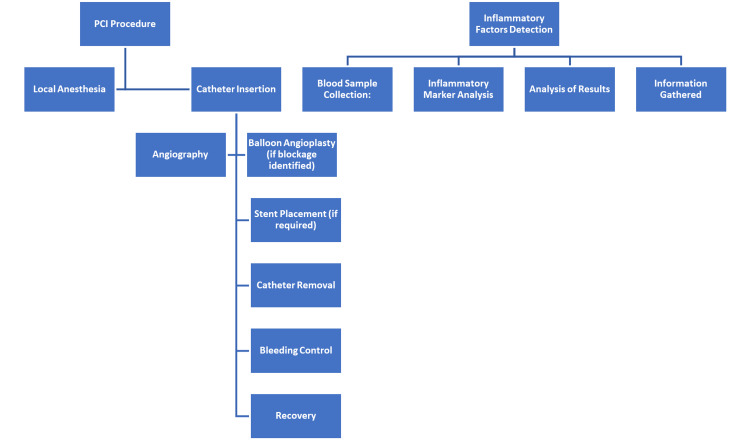
Flowchart showing the methods for surgery and detection PCI: percutaneous coronary intervention

Procedures for PCI involve several steps to enhance coronary blood supply, restore myocardial blood flow, and alleviate symptoms associated with CVEs (Figure 1). Initially, the patient undergoes local anesthesia to numb the area, followed by insertion of a guidewire into the coronary artery system via a femoral or radial artery puncture. Subsequently, a contrast agent is injected to visualize any arterial narrowings or blockages. Using a balloon, the narrowed artery is dilated to restore blood flow, and then a stent is implanted to maintain vessel patency. Post-implantation, the guidewire and catheter are removed, and pressure may be applied to the external insertion site to manage bleeding. Patients are then monitored in the recovery room for any complications. PCI, a widely employed procedure, effectively treats CVEs by improving blood flow to the heart muscle, thereby alleviating symptoms and enhancing patient prognosis. Following treatment, inflammatory factors are detected by collecting fasting blood samples and utilizing assays such as enzyme-linked immunosorbent assay (ELISA) for C-reactive protein (CRP) and tumor necrosis factor-α (TNF-α), along with chemiluminescent immunoassay (CLIA) for procalcitonin (PCT). These tests provide crucial insights into post-intervention inflammatory and infectious statuses, aiding in patient care.

Statistical analysis

We have conducted a power analysis using G Power software to assess the statistical power of our study. The analysis revealed that with our sample size and effect size estimates, our study had adequate power (>80%) to detect significant differences in the primary outcomes between the PPI and Non-PPI groups. Data were statistically analyzed using SPSS 22.0 (IBM Corp., Armonk, NY, USA). Continuous variables with a normal distribution were displayed as mean ± standard deviation and analyzed using t-tests. Categorical data were presented as percentages (%) and analyzed with the chi-square test. P<0.05 was utilized to determine statistical significance.

Ethical statement

This is a retrospective study, which excluded and screened relevant cases according to research criteria, and was conducted in accordance with the 1964 Declaration of Helsinki and its later amendments or similar ethical standards. Informed consent exemption was obtained due to the retrospective nature of this study.

## Results

Among these patients, 91 received PPI treatment, and 75 (82.42%) did not receive PPI treatment (Table 1). The PPI group enrolled 62 male (67.03%) and 29 female (31.51%) patients, with ages ranging from 28 to 92 years (59.73 ± 10.68 years old on average). The average body mass index (BMI) value in this group was 24.17 ± 4.21. In the non-PPI group, there were 54 male (72%) and 21 female (28%) patients, who were 29 to 85 (57.33 ± 11.26) years old, with an average BMI value of 24.59 ± 4.18. In comparing the Non-PPI and PPI groups, similar prevalence rates were observed for drinking history (58.00% vs. 57.00%) and smoking history (60.00% vs. 61.00%). Additionally, both groups exhibited a high prevalence of submerged ulcers (94.00% vs. 95.00%). Regarding comorbidities, the Non-PPI group had slightly higher rates of diabetes (84.00% vs. 82.00%) and hypertension (49.00% vs. 46.00%) compared to the PPI group. Comparisons of age, gender, prevalence of hypertension, diabetes, peptic ulcers, smoking history, and alcohol consumption history of patients in different groups exhibited no observable differences (P > 0.05), indicating that the groups were comparable in terms of these characteristics from different groups.

**Table 1:** Comparison of basic data of patients PPI: proton pump inhibitors

Group		PPI Treatment	No PPI Treatment
Patients		91	75
Gender	Male	62 (67.03%)	54 (72%)
	Female	29 (31.51%)	21 (28%)
Age (years)		59.73 ± 10.68	57.33 ± 11.26
BMI		24.17 ± 4.21	24.59 ± 4.18
Drinking History (%)		52 (57.00%)	43 (58.00%)
Smoking History (%)		56 (61.00%)	45 (60.00%)
Submerged Ulcers (%)		86 (95.00%)	71 (94.00%)
Diabetes (%)		75 (82.00%)	63 (84.00%)
Hypertension (%)		42 (46.00%)	37 (49.00%)
P-value (comparisons)		>0.05	

Incidence of UGIB in patients

Melena was the predominant clinical manifestation, with 129 cases (77.71%), followed by 24 cases (14.46%) of hematemesis accompanied by melena, and 13 cases (7.83%) with no overt clinical bleeding symptoms. The distribution of anticoagulant types in the PPI group includes 75 patients receiving aspirin and 24 receiving clopidogrel, while in the Non-PPI group, 54 patients received aspirin and 21 received clopidogrel. A history of previous GI bleeding was noted in 10 patients (12.35%) in the PPI group and five patients (6.17%) in the Non-PPI group (Table 2).

**Table 2:** Incidence of upper gastrointestinal bleeding (UGIB) among patients receiving dual antiplatelet therapy (DAPT), classified by clinical presentation and anticoagulant type in proton pump inhibitor (PPI) and Non-PPI groups Dosage: Aspirin (100 mg/d), Clopidogrel (75 mg/d)

Clinical Presentation	Total Number of Cases	Percentage (%)	PPI Group (N)	Non-PPI Group (N)	Previous History of GI Bleeding (Number of Patients)	Previous History of GI Bleeding (%)
Melena	129	77.71	Aspirin (75), Clopidogrel (24)	Aspirin (54), Clopidogrel (21)	10	12.35
Hematemesis with Melena	24	14.46	Aspirin (10), Clopidogrel (14)	Aspirin (14), Clopidogrel (10)	5	6.17
No Overt Clinical Symptoms	13	7.83	-	-	3	3.70

Incidence of CVEs in patients from various groups

The incidence rates (IR) of CVEs among patients in the PPI treatment group and the Non-PPI treatment group were 19.78% and 20% respectively (Table 3). In the PPI treatment group, out of 91 patients, 18 experienced CVEs, yielding an incidence rate of 19.78%. Conversely, in the Non-PPI treatment group, out of 75 patients, 15 experienced CVEs, resulting in an incidence rate of 20%. Upon analyzing the breakdown of CVEs, it's evident that the distribution of specific events varied between the groups. Notably, in the PPI group, a higher percentage of cases comprised angina (16.67%) and acute coronary syndrome (ACS) (38.89%) compared to the Non-PPI group where these percentages were lower (4% and 6.67% respectively). Despite these differences in event distribution, statistical analysis indicated no significant difference in CVE incidence rates between the PPI and Non-PPI treatment groups (P>0.05). Therefore, while the specific types of CVEs varied between the groups, the overall incidence rates of these events did not demonstrate a statistically significant difference, suggesting that the use of PPIs does not significantly impact the risk of experiencing cardiovascular events compared to non-PPI treatments.

**Table 3:** Comparison of cardiovascular event (CVE) incidence rates and breakdown by event type in proton pump inhibitor (PPI) and Non-PPI treatment groups MI: myocardial infarction, ACS: acute coronary syndrome, HF: heart failure

		PPI Treatment Group	Non-PPI Treatment Group
Total Patients		91	75
Cases with CVEs		18	15
Incidence Rate (IR)		19.78%	20%
CVEs Breakdown n (%)	Angina	3 (16.67%)	3 (4%)
	MI	3 (16.67%)	2 (2.67%)
	Stent Thrombosis	1 (5.56%)	1 (1.33%)
	ACS	7 (38.89%)	5 (6.67%)
	Stroke	1 (5.56%)	1 (1.33%)
	HF	2 (11.11%)	2 (2.67%)
	Death	1 (5.56%)	1 (1.33%)
PPI vs. Non-PPI IR		P>0.05	

Distribution of CVEs induced by different PPI drugs

**Figure 2:**
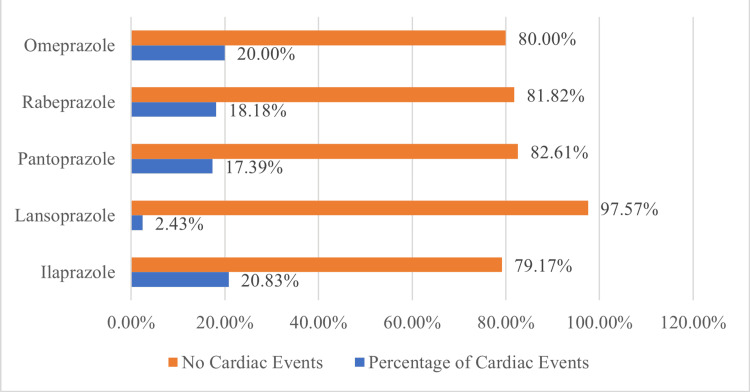
Incidence rates (IRs) of cardiovascular events (CVEs) in each subgroup of proton pump inhibitors (PPIs)

Based on different types of PPIs used, 91 patients were further grouped into five: esomeprazole, lansoprazole, pantoprazole, rabeprazole, and omeprazole, with five, six, four, two, and one cases suffering from CVEs, respectively. The resulting IRs of CVEs are demonstrated in Figure 2. Statistical analysis revealed no considerable difference in IRs of CVEs among the various PPI subgroups (P>0.05).

Comparison of inflammatory indicators

**Figure 3:**
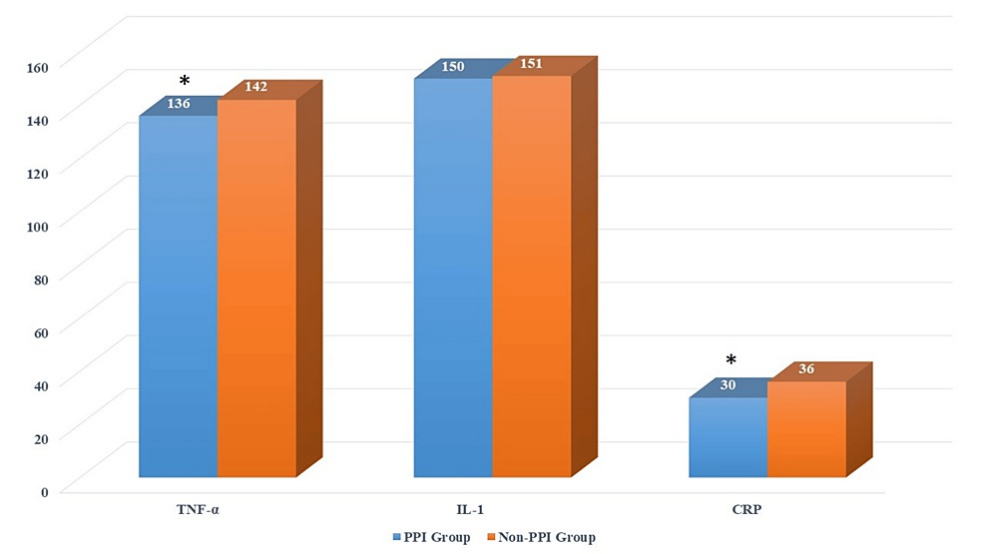
Changes in TNF-α, IL-1 and CRP of patients receiving proton pump inhibitors (PPI) and no PPI. Note: * suggested a great difference with P<0.05 TNF-α: tumor necrosis factor-α, IL-1: interleukin-1, CRP: C-reactive protein

In comparing the PPI and Non-PPI groups, the levels of inflammatory markers differed marginally. The PPI group exhibited slightly lower levels of TNF-α (136 ng/L vs. 142 ng/L) and interleukin-1 (IL-1) (150 ng/L vs. 151 ng/L) compared to the Non-PPI group. However, the CRP levels were notably lower in the PPI group compared to the Non-PPI group (30 mg/L vs. 36 mg/L). These findings suggest that while there are subtle variations in TNF-α and IL-1 levels between the groups, the CRP levels significantly differ, indicating a potential influence of PPI on inflammation. The average levels of CRP and TNF-α in the PPI group were significantly lower compared to those in the non-PPI group, showing significant differences with P<0.05. Meanwhile, IL-1 exhibited no observable difference for patients between the PPI and Non-PPI groups (P>0.05). The above results are explicated in Figure 3.

## Discussion

CVEs refer to diseases or pathological conditions involving the heart and vascular system, including MI, angina, coronary heart disease, stroke, thrombosis, and others [13]. These events are often caused by factors such as vascular narrowing, thrombus formation, or cardiac dysfunction, significantly affecting patients' quality of life and survival rates [14]. In the management of cardiovascular diseases (CVDs), DAPT, PPIs, and inflammatory factors play crucial roles [15].

DAPT refers to the simultaneous use of two antiplatelet drugs to prevent the occurrence of CVEs [16]. The commonly applied DAPT regimen includes the combination of aspirin and clopidogrel. Aspirin inhibits the COX-1 enzyme in platelets, blocking the synthesis of TXA2, a potent platelet aggregating agent, thus reducing the risk of CVEs. Aspirin also interferes with platelet adhesion to the vessel wall, reducing platelet deposition and thrombus formation on damaged vessel walls, thereby lowering the risk of CVEs [17,18]. Clopidogrel, on the other hand, works by binding to the P2Y12 receptor, blocking the action of ADP, and reducing the risk of platelet aggregation and thrombus formation. DAPT has been extensively utilized in managing CVDs and has been shown to significantly reduce the incidence of cardiac events [19]. However, DAPT is also associated with some side effects, one of which is UGIB [20]. The results of this study indicate that melena is the most common gastrointestinal clinical symptom in patients who develop UGIB while on DAPT. This finding aligns with previous research and clinical experience. Therefore, clinical practitioners can promptly detect and manage UGIB by monitoring patient symptoms and conducting routine tests, such as fecal occult blood tests, to reduce unnecessary complications and risks.

The use of antiplatelet drugs can impact platelet function, potentially leading to gastrointestinal mucosal damage and bleeding. To reduce the risk of UGIB, clinicians often choose to combine the use of PPIs [21]. PPIs work by inhibiting the activity of the proton pump responsible for stomach acid secretion, thus reducing acid production. By binding to the proton pump enzyme, PPIs effectively decrease gastric acid secretion and release, protecting the upper gastrointestinal mucosa from irritation and damage caused by antiplatelet therapy, thereby reducing the risk of bleeding [22,23]. This work demonstrated that concurrent use of PPIs with DAPT in patients undergoing PCI did not increase the occurrence of CVEs. It suggests that PPI usage not only reduces the risk of UGIB but also does not negatively impact the cardiovascular health of patients [24]. These results provide some safety assurance for the combined use of PPIs and DAPT in PCI patients and can guide clinical decision-making for healthcare professionals. Overall, using PPIs in combination with DAPT after PCI appears to be a safe approach, offering protection against UGIB while not adversely affecting cardiovascular health. This insight can help guide clinicians in their treatment decisions for patients after PCI procedures.

Furthermore, inflammatory factors play a crucial role in the development and progression of CVDs [25]. Research has shown that the inflammatory response is closely related to pathological processes such as endothelial dysfunction, plaque formation and rupture, and thrombosis [26,27]. Results in this work indicated that the concurrent use of PPIs with DAPT in patients after PCI can lower the levels of inflammatory factors in the serum of patients. This may be due to the anti-inflammatory properties of PPIs, which can alleviate vascular wall inflammation, improve vascular function, and further reduce the occurrence of CVEs [28]. This finding implies that the combined use of PPIs may have certain anti-inflammatory effects and may positively regulate the inflammatory process of CVDs [29]. Inflammatory response is critical in the progression of CVDs, and thus, reducing inflammatory factor levels can have a positive impact on disease prognosis [30]. However, further research is needed to confirm the specific mechanisms of PPIs in regulating the inflammatory response and their potential value in clinical practice.

The limitations of this study are that our analysis relies on a relatively small sample size, which may limit the generalizability of our findings. Additionally, as with any retrospective analysis, there exist potential biases inherent in the study design that could influence the observed associations. We recognize the importance of controlling for confounding variables, and while efforts were made to address this through statistical adjustments, the possibility of residual confounding remains. The absence of a control group not receiving dual antiplatelet therapy or PPI treatment limits our ability to draw definitive conclusions about the effects of these interventions independently. Future prospective studies with larger sample sizes and comprehensive data collection are warranted to confirm and extend our findings, ultimately informing clinical practice more effectively.

## Conclusions

CVEs represent a serious group of diseases that pose a threat to patients' health and life. DAPT, as a frequently used treatment regimen, greatly reduces the incidence of CVEs. However, UGIB is one of its common side effects, and the concurrent use of PPIs can reduce the risk of UGIB. Moreover, inflammatory factors closely associate with progression of CVDs, and PPIs may have a protective effect on CVEs by reducing inflammatory factor levels. The findings in this work offered important support for the application of DAPT in the management of CVDs. They highlight the need for clinicians to pay greater attention to the recognition and management of UGIB symptoms and support the safety and effectiveness of combined PPI and DAPT use in patients after PCI. These conclusions need further research for validation and refinement to maximize treatment efficacy and prognosis for patients, better guide clinical practice, and improve patient outcomes.
